# Tumor-like characteristics of vascular smooth muscle cells in atherosclerosis

**DOI:** 10.3389/fphar.2025.1734123

**Published:** 2026-01-02

**Authors:** Xiangchun Li, Yuyan Deng

**Affiliations:** Department of Critical Care Medicine, Weifang People’s Hospital, Weifang, Shandong, China

**Keywords:** atherosclerosis, phenotypicplasticity, tumor-like characteristics, vascular smooth muscle cell-derived cells, vascular smooth muscle cells

## Abstract

Atherosclerosis is a complex, chronic inflammatory disease, traditionally considered a lipid-driven and immune-mediated disorder whose pathogenesis involves the interplay of multiple cellular populations. Vascular smooth muscle cells (SMCs) have long been considered contractile and extracellular matrix-producing cells that stabilize the fibrous cap. However, recent advances in lineage tracing and single-cell transcriptomics have revealed that vascular SMCs possess a high degree of plasticity and exhibit tumor-like characteristics during atherogenesis. Vascular SMCs and vascular smooth muscle cell-derived cells (SDCs) in atherogenesis exhibit DNA damage and genomic instability, evade senescence, hyperproliferate, activation of migration and invasion, cancer stem cell-like property, and activate multiple cancer-related signaling pathways. These tumor-like behaviors accelerate phenotypic switching of vascular SMCs and lesion progression. In this review, we mainly discuss the tumor-like characteristics of vascular SMCs, aiming to provide a theoretical basis for new therapeutic strategies targeting vascular SMCs and provide new ideas for the precise prevention and treatment of atherosclerosis.

## 1 Introduction

Atherosclerosis is a chronic disease that primarily affects medium- and large-sized arteries, causing stenosis or even complete occlusion of the vascular lumen (Azar et al., 2025). It is a major cause of cardiovascular events such as angina, myocardial infarction, ischemic stroke, and peripheral arterial disease (Makover et al., 2022; Mlynarska et al., 2024). This lesion typically develops insidiously within the arterial intima over decades, ultimately forming a complex atheromatous plaque (Madaudo et al., 2024). Plaques contain a diverse array of cells, including inflammatory cells, immune cells, foam cells, endothelial cells, and vascular smooth muscle cells (SMCs) (Basatemur et al., 2019; Thazhathveettil et al., 2024), whose behavior is regulated by the local microenvironment, intercellular signaling, and spatial proximity (Zhao et al., 2025). For decades, endothelial dysfunction, lipid deposition, macrophage infiltration, and chronic inflammation have been regarded as the primary drivers of plaque formation (Kong et al., 2022), whereas the fundamental role of SMCs in lesion development has been largely underappreciated. Increasing studies have demonstrated that SMCs are the most abundant cell type in most advanced atherosclerotic plaques and possess a high degree of plasticity, capable of undergoing phenotypic transitions in response to various stimuli, exhibiting diverse states including synthetic, inflammatory, macrophage-like, and fibrocartilaginous (Basatemur et al., 2019; Cao et al., 2022). However, the association between these properties of SMCs and the pathogenesis of atherosclerosis remains unclear.

Previous studies have suggested potential mechanistic similarities between atherosclerosis and cancer. Studies have shown that certain SMC subpopulations within the arterial media can proliferate in a clonal manner, with patterns highly resembling the clonal expansion of tumor cells within lesions (Tapia-Vieyra et al., 2017; Raposeiras Roubin and Cordero, 2019). Notably, a recent study by Pan et al. (Pan et al., 2024) demonstrates that smooth muscle cell-derived cells (SDCs) exhibit cancer-like characteristics, including genomic instability, bypass of cellular senescence, sustained proliferation and survival, enhanced migration and invasion, and cancer stem cell-like properties (Pan et al., 2024). Additionally, a review paper by Steffensen et al. reported that human carotid atherosclerotic plaques frequently contain somatic mutations, as shown by whole-exome sequencing, whereas non-atherosclerotic arteries do not (Steffensen et al., 2025). Many plaques harbor multiple mutated cellular clones, some representing over 10% of the total cell population and often involving genes related to the contractile machinery (Steffensen et al., 2025). These findings indicate that clonal expansion is a common feature of human atherosclerosis, involving both locally derived and circulating mutated cells.

Therefore, tumor-like programmed behaviors of SMCs may provide new insights into the biological nature of plaques. Building on existing research, this article will systematically explore the tumor-like characteristics exhibited by SMCs and SDCs. These findings will help deepen our understanding of the plaque formation process and thus provide innovative directions for the understanding and treatment of atherosclerosis and related cardiovascular diseases.

## 2 Tumor-like characteristics of vascular smooth muscle cells

**FIGURE 1:**
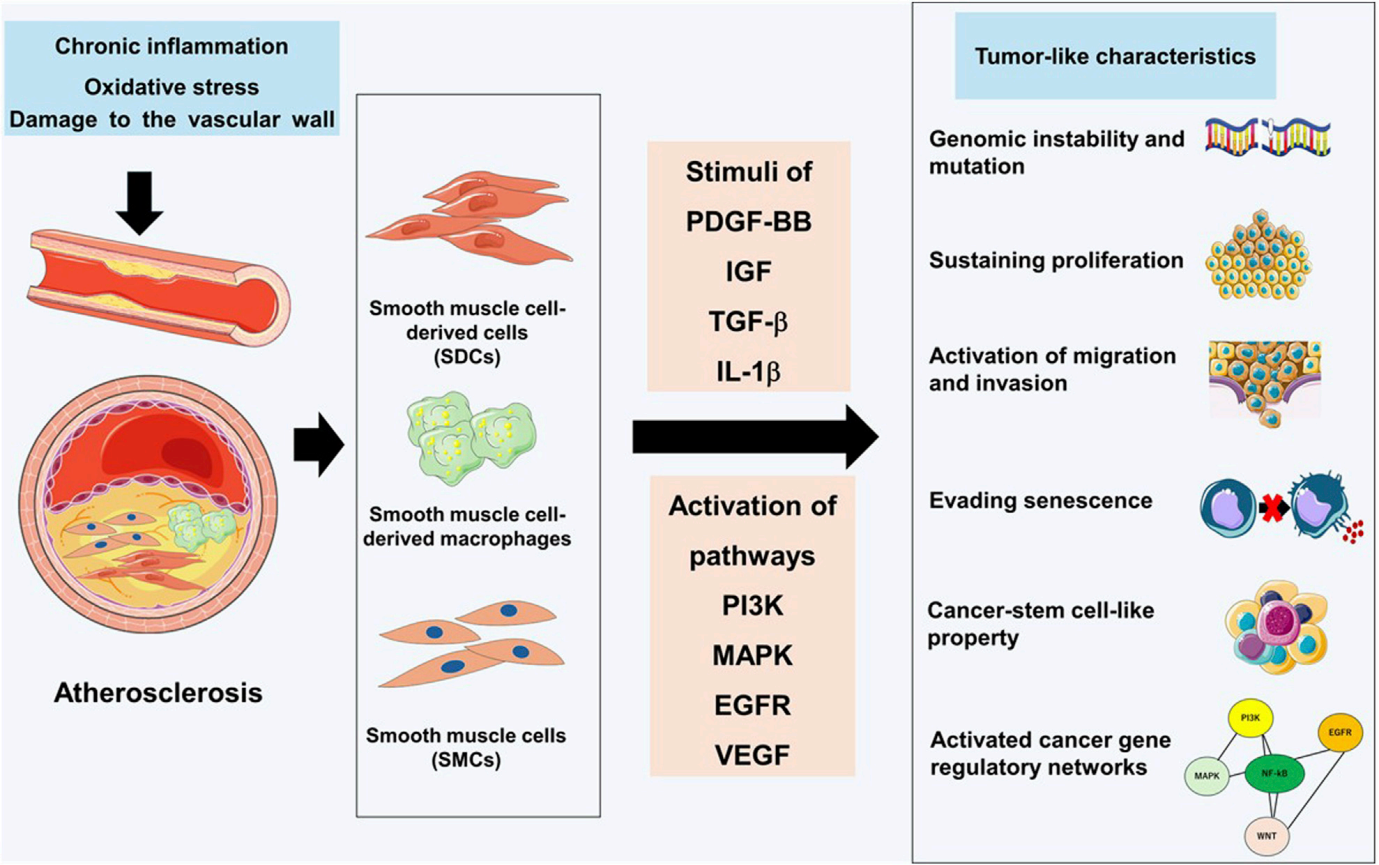
Tumor-like characteristics of vascular smooth muscle cells in atherosclerosis. Following vascular wall injury, the release of stimulating factors such as PDGF-BB, IGF, TGF-β, and IL-1β activates multiple signaling pathways including PI3K, MAPK, EGFR, and VEGF. Under the influence of these signaling pathways, vascular smooth muscle cells (SMCs) and smooth muscle cell-derived cells (SDCs) in atherosclerosis exhibit various tumor-like characteristics, including genomic instability and mutations, persistent proliferation, enhanced migration and invasion abilities, senescence evasion, cancer stem cell-like characteristics, and activation of cancer gene regulatory networks. These tumor-like features can accelerate phenotypic transformation of SMCs and exacerbate the progression of atherosclerosis.

Human genetic studies, combined with single-cell analysis and SMCs lineage tracing strategies, have revealed that SMCs and SDCs constitute the primary cellular components of atherosclerosis (Bentzon and Majesky, 2018; Sharma et al., 2024). The emergence of lineage-tracing and transcriptomic studies has demonstrated that VSMCs comprise a much larger proportion of atherosclerotic plaques than originally thought, demonstrate multiple different phenotypes *in vivo*, and have roles that might be detrimental (Grootaert and Bennett, 2021). Recent studies have redefined atherosclerosis as a tumor-like process driven in part by vascular SMCs (Pan et al., 2024). These tumor-like behaviors accelerate vascular SMCs phenotypic transformation and disease progression (Figure 1). Then, we discuss in detail these tumor-like behaviors of SMCs in atherosclerosis.

### 2.1 Genomic instability

Genomic instability is a core characteristic of tumor cells and a key driver of cancer development and progression, including gene mutations, chromosomal abnormalities, and other genetic alterations (Negrini et al., 2010; Requesens et al., 2024). Studies have found that in atherosclerosis, vascular SMCs exhibit significant genomic instability, which is associated with increased oxidative stress and defects in DNA repair mechanisms (Shah and Mahmoudi, 2015; Zha et al., 2022). During the early phenotypic transition of atherosclerosis, SMCs within plaques undergo extensive DNA damage (Grootaert and Bennett, 2021), predominantly in the form of oxidative stress-induced lesions. This damage accumulates disease progression, particularly in SDCs and within the intimal region. In advanced atherosclerotic plaques, numerous single- and double-strand DNA breaks are observed (Shah and Bennett, 2016), ultimately leading to genomic instability.

A study by Gray et al. (2015) demonstrated that human atherosclerotic plaque VSMCs exhibit substantial DNA damage and activation of double-stranded breaks (DSB) repair pathways, yet altering DSB repair capacity does not modify the overall progression of atherosclerosis. Recently, Pan et al. analyzed single-cell transcriptome data from mouse and human atherosclerotic plaques and found that nearly all cell types, particularly SMC-derived cells, exhibit extensive large-scale chromosomal copy number variation (Pan et al., 2024). These findings suggest that SMCs gradually accumulate significant genomic instability during phenotypic transitions. Notably, SDCs exhibit increased formation of micronuclei resulting from chromosomal fragmentation, further indicating that these cells harbor severe genomic instability (Pan et al., 2024).

### 2.2 Evasion of senescence

Senescence functions as a protective barrier against uncontrolled proliferation. Senescence-associated β-galactosidase (SA-β-Gal) is nearly undetectable in arteries of young, non-atherosclerotic mice but significantly elevated in the medial layer of SMCs in aged mice, which also express senescence markers, including SA-β-Gal, p21, and p16 (Pan et al., 2024). Within atherosclerotic lesions, the majority of lesional cells, including SDCs, are negative for SA-β-Gal, with only some cells in the media, fibrous cap, and shoulder regions expressing SA-β-Gal. Further experiments *in vitro* showed that SMCs gradually acquire a senescent phenotype during serial passage, whereas SDCs rarely express SA-β-Gal even after prolonged culture and significantly lower levels of p21 and p16 compared with SMCs (Pan et al., 2024). These findings suggest that SDCs can, to some extent, escape replicative senescence, thereby maintaining a strong proliferative capacity.

### 2.3 Sustaining proliferation

During the progression of atherosclerosis, vascular SMCs transform from a quiescent contractile phenotype to a synthetic phenotype with synthetic and proliferative activity (Chen et al., 2023). This transition is reflected not only in their increased proliferative and migratory capacity, but also in the extensive synthesis and deposition of extracellular matrix. Unlike the transient and tightly regulated proliferation observed during normal vascular repair, vascular SMCs within atherosclerotic plaques remain in a persistently activated and hyperproliferative state, driven by chronic inflammatory stimuli, lipid deposition, and various growth factors (Zhao et al., 2025). Such abnormal and sustained proliferative behavior bears resemblance to tumor-like cell growth and is frequently accompanied by both genetic and epigenetic alterations. Single-cell RNA sequencing studies have revealed the presence of a distinct subpopulation of “primed” vascular SMCs in healthy vessels. They have atypical transcriptional profiles that are highly similar to activated cells detected in experimental atherosclerosis models and human lesions (Dobnikar et al., 2018). These cells show significantly enhanced proliferation potential, suggesting that vascular SMCs are not passive responses in the process of atherosclerosis, but there is a potential precursor subpopulation that can be activated by the pathological environment. Another study found that SDCs in atherosclerotic lesions have a significantly higher proliferation rate than normal vascular SMCs. More strikingly, SDCs not only possess a sustained proliferative capacity but also maintain stable and vigorous division activity during long-term *in vitro* culture, without experiencing proliferation decline (Pan et al., 2024). This is highly similar to the high division potential of typical tumor cells. Furthermore, multiple key signaling pathways associated with tumor cell proliferation are activated in the abnormal proliferation of vascular SMCs, including the PI3K/AKT and MAPK/ERK pathways (Mehrhof et al., 2005), and the Notch and Wnt/β-catenin pathways (Azhdari and Zur Hausen, 2025). Collectively, the abnormal proliferation of vascular SMCs in atherosclerosis not only exhibits uncontrolled characteristics similar to those of tumor cells, but also shares a high degree of overlap with tumor proliferation in terms of molecular mechanisms and signaling pathways.

### 2.4 Invasiveness property

During atherosclerosis, vascular SMCs exhibit invasive behavior similar to tumor cells. Transitioning from a contractile to a synthetic phenotype, they gain migratory and proliferative abilities similar to tumor cells undergoing malignant transformation. By secreting matrix metalloproteinases, vascular SMCs degrade the extracellular matrix and internal elastic lamina (Wang and Khalil, 2018), enabling infiltration of plaques much like tumor invasion of surrounding tissues. Within lesions, vascular SMCs contribute to fibrous cap stability but also transdifferentiate into macrophage-like cells, driving inflammation and necrotic core expansion (Luo et al., 2023). Single-cell sequencing has revealed that many inflammatory-like cells in plaques originate from these infiltrating vascular SMCs, paralleling tumor-stromal interactions (Pan et al., 2024). Thus, vascular SMC invasion is a central driver of atherosclerosis progression and represents a biological parallel to tumor cell invasion.

### 2.5 Cancer stem cell-like property

Within atherosclerotic lesions, some vascular SMC subpopulations exhibit caner stem cell-like properties, possessing self-renewal and multipotential differentiation potential, enabling them to play a dynamic role in the progression of atherosclerosis. Studies have shown that approximately 0.15% of SDCs in serum-free suspension culture can form three-dimensional spheroids, displaying typical characteristics of cancer stem cells (Pan et al., 2024). Furthermore, SDCs derived from atherosclerotic plaques significantly express cancer stem cell-associated marker genes, such as CD24 and CD44 (Pan et al., 2024). Overall, these results suggest that during atherogenesis, SMCs undergo a phenotypic shift, acquiring a stem cell-like property similar to tumor cells.

Although several studies have proposed the presence of cancer stem cell-like SDCs within atherosclerotic lesions, the supporting evidence remains limited and is largely derived from *in vitro* observations or lineage-tracing experiments in animal models. Direct *in vivo* confirmation-particularly in human plaques-remains scarce. Most reported stem-like properties rely on culture-induced behaviors, which may not fully reflect the native plaque environment. Therefore, while the concept of cancer stem cell-like-like SDCs is intriguing, current data should be interpreted with caution until more robust *in vivo* and human-tissue evidence becomes available.

### 2.6 Activated cancer gene regulatory networks

In human atherosclerotic plaques, Pan et al. inferred and validated changes in the activity of cancer-related signaling pathways based on single-cell transcriptome data from mouse atherosclerosis. Their results revealed that compared with SMCs, SDCs showed significant activation of multiple cancer signaling pathways, including p53, PI3K, EGFR, TNFα, NFκB, MAPK, WNT, and VEGF signaling pathways (Pan et al., 2024). Furthermore, the proportion of cells with multiple pathways activated was significantly increased. Increased p53 pathway activity suggests a heightened DNA damage response and genomic instability in SDCs (Yeo et al., 2016). Activation of the TNFα and NFκB pathways enhance SDC survival (Lamb et al., 2020). Activation of the PI3K, MAPK, EGFR, and VEGF pathways are closely associated with significantly enhanced SDC proliferation, with PI3K potentially contributing to invasiveness (Panda et al., 2025). Finally, activation of the WNT pathway drives SDCs to acquire cancer stem cell-like renewal properties (Xue et al., 2025). These results indicate that the phenotypic transformation of SMCs to SDCs is not only accompanied by abnormal activation of multiple cancer signaling pathways, but also confers tumor-like characteristics to SDCs, thus revealing the deep commonalities between atherosclerosis and tumors in molecular mechanisms.

## 3 Discussion

### 3.1 Tumor-like characteristics of VSMCs: driver or consequence?

Recent studies have revealed that VSMCs within atherosclerotic plaques frequently exhibit genomic instability (Coll-Bonfill et al., 2023), somatic mutations (Revechon et al., 2023), and clonal expansion (Wang et al., 2019), raising the question of whether these “tumor-like” features act as drivers of atherosclerosis or merely represent consequences of chronic inflammation and oxidative stress. Increasing evidence suggests that both interpretations capture important aspects of the disease. On one hand, the atherosclerotic microenvironment is characterized by sustained inflammation, high reactive oxygen species (ROS) levels, and impaired DNA repair capacity (Nowak et al., 2017). These factors can induce DNA strand breaks, base oxidation, and epigenetic reprogramming in VSMCs, leading to the accumulation of mutations and promoting the selection of proliferative, apoptosis-resistant subclones. This view positions genomic instability largely as a secondary response to environmental stressors. On the other hand, emerging sequencing and lineage-tracing data indicate that certain VSMC-derived clone s harbor mutations or transcriptional programs that confer growth or survival advantages, enabling them to expand disproportionately and actively shape plaque progression. These cells often display enhanced proliferation, migratory behavior, extracellular matrix remodeling, and resistance to cell death-features reminiscent of neoplastic processes. In some experimental models, introducing oncogene-like alterations in VSMCs accelerates lesion development, supporting a more direct driver role for tumor-like phenotypes.

Inflammation and oxidative injury initiate DNA damage and phenotypic reprogramming, while genomically altered VSMC clones subsequently amplify local inflammation, matrix degradation, and plaque expansion. This phenomenon explains why the tumor-like behavior of VSMCs does not progress to true malignancy yet significantly contributes to disease evolution. Understanding how environmental stressors and intrinsic genetic changes interact to govern VSMC fate may offer new therapeutic opportunities-ranging from limiting oxidative DNA damage to selectively targeting pathogenic clonal expansions-ultimately providing a more precise strategy for slowing atherosclerotic progression.

### 3.2 Clinical and therapeutic implications

In the study of atherosclerosis, the tumor-like behavior of vascular SMCs has not only revealed novel pathogenesis but also offered potential clinical and therapeutic implications. First, identifying tumor-like behavior of SMCs as a novel therapeutic target provides new insights into the prevention and treatment of atherosclerosis. Studies have shown that niraparib, a clinical anticancer drug, reduces plaque burden, increases fibrous cap thickness, and reduces necrotic cores in mice with atherosclerosis (Pan et al., 2024). In addition, intervening in classic oncogenic signaling pathways has the potential to effectively inhibit abnormal vascular SMC proliferation, thereby delaying disease progression. Second, genomic alterations and abnormal activation of signaling pathways could serve as biomarkers for identifying aggressive disease subtypes, providing a basis for early diagnosis and personalized treatment. Furthermore, the striking parallels between abnormal proliferation of vascular SMCs and tumorigenesis suggest that insights from oncological research and even the direct repurposing of certain anticancer agents may offer promising new avenues for cardiovascular therapy. Additionally, combining vascular SMCs-targeted intervention with traditional lipid-lowering and anti-inflammatory treatments may produce synergistic effects, not only slowing disease progression but also potentially playing a greater role in reducing recurrence rates and improving prognosis. Overall, these clinical and therapeutic explorations provide broad space for future translational medicine research and lay a theoretical foundation for the development of innovative cardiovascular treatments.

### 3.3 Future perspectives

Several critical scientific questions remain unresolved in the study of vascular SMC tumorigenesis. First, are genomic alterations the primary drivers of phenotypic transformation, or merely consequences of the inflammatory microenvironment? Second, what specific roles do distinct SDC subsets play in fibrous cap thinning and repair? Third, can short-term, precisely timed interventions preserve vascular homeostasis while durably reshaping VSMC fate? Finally, which biomarkers can reliably detect pathological changes in VSMCs at the earliest stages of disease? Addressing these questions will be essential for translating mechanistic insights into clinically viable strategies for prevention and therapy.

In the development of atherosclerosis, vascular SMCs extend their roles beyond their traditional contractile and matrix-forming functions. Tumor-like programs are activated, including genomic instability, evasion of senescence, sustained proliferation and survival, activated migration and invasion, cancer stem cell-like properties, and activated tumor-associated gene regulatory networks. Recognizing vascular SMCs as malignant tumor-like players not only shifts the paradigm of understanding the pathogenesis of atherosclerosis but also reveals new opportunities for therapeutic innovation.
